# The Nance-Horan syndrome protein NHS regulates cell migration persistence by organizing WAVE and N-WASP nucleation-promoting factor complexes

**DOI:** 10.1016/j.jbc.2026.113443

**Published:** 2026-08-13

**Authors:** Ekaterina Tsydenzhapova, Artem I. Fokin, Nicolas B. David, Nathalie Rocques, Anna Polesskaya, Iman Haddad, Joëlle Vinh, Raphaël Guérois, Alexis M. Gautreau

**Affiliations:** 1 Laboratoire de Biologie Structurale de la Cellule, BIOC, CNRS, École Polytechnique, Institut Polytechnique de Paris, Palaiseau, France; 2 Laboratoire d’Optique et Biosciences, LOB, CNRS, École Polytechnique, Institut Polytechnique de Paris, Palaiseau, France; 3 Biological Mass Spectrometry and Proteomics (SMBP), ESPCI Paris, Université PSL, CNRS UAR2051, Paris, France; 4 Université Paris-Saclay, CEA, CNRS, Institute for Integrative Biology of the Cell (I2BC), Gif-sur-Yvette, France

**Keywords:** cell migration, Nance-Horan syndrome, WAVE Regulatory complex, WAVE Shell complex, N-WASP/WIPF2 complex, ABL2, Arp2/3 complex, mass spectrometry

## Abstract

The function of the *NHS* gene that is responsible for Nance-Horan Syndrome has remained elusive at the cellular level. Using CRISPR/Cas9, we inactivated the *NHS* gene in MCF10A cells and characterized the isoforms expressed in these cells. *NHS* KO cells displayed reduced migration persistence, a phenotype that was fully rescued by the long isoform 1 (i1) that contains an N-terminal WAVE Homology Domain (WHD), but only partially rescued by the short isoform two (i2), which does not. Patient mutations resulting in NHS proteins truncated at their C-terminus also reduced the ability of NHS i1 to rescue migration persistence. Using Tandem Affinity Purification (TAP) of NHS i1 and mass spectrometry, we identified as major NHS partners all subunits of the WAVE Regulatory Complex (WRC) except WAVE subunits themselves, indicating that the WHD of NHS assembles a WAVE Shell Complex (WSC). The Arp2/3 complex and the Nucleation Promoting Factor (NPF) WAVE that activates it are critical for migration persistence. To investigate the role of the NHS-containing WSC, we performed TAP of the ABI1 subunit in parental and *NHS* KO cells and identified differential partners associated with ABI1 only in parental cells, but not in *NHS* KO cells. The most abundant of these were the WIPF2/N-WASP complex, which together with the kinase ABL2, was also critical for migration persistence. These results suggest that NHS controls cell migration by remodeling NPF complexes and their higher order assembly.

The Nance-Horan Syndrome is a genetic disorder characterized by congenital cataracts associated with dental anomalies, a subtly dysmorphic face and sometimes developmental delay. The causative gene, *NHS*, has been identified on the X chromosome (1, 2). Heterozygous females carrying a single mutated *NHS* allele display a milder phenotype than hemizygous males (3, 4). Most mutations found in patient families result in a truncated NHS protein due to nonsense mutations or frameshifts caused by small deletions (5, 6, 7, 8, 9, 10, 11, 12). Copy number variations of the wild-type *NHS* gene suffice to yield isolated cataracts in the absence of other symptoms (7).

The *NHS* gene produces several protein isoforms through alternative promoters and an alternatively spliced exon (13). The longest isoform, referred to as NHS-A, localizes to the plasma membrane of epithelial cells, whereas a shorter isoform, referred to as NHS-1A, stemming from another promoter and therefore having a different N-terminus is cytosolic. When the long isoform harbors patient mutations, the truncated NHS protein is also cytosolic, indicating that both N- and C-terminal parts of the protein are required for the membrane localization of NHS (14). NHS localizes to cell-cell junctions, perhaps through its interaction with the tight junction protein ZO-1 (15). NHS has also been reported to localize at focal adhesions and lamellipodia (16).

Lamellipodia are adhesive membrane protrusions that develop at the front of migrating cells through polymerization of branched actin (17). When activated by the Nucleation Promoting Factors (NPFs) WAVE and N-WASP, the Arp2/3 complex generates branched actin that powers membrane protrusions. WAVE and N-WASP are themselves tightly regulated within the pentameric WAVE Regulatory Complex (WRC) and the dimeric complex of N-WASP with WIP family proteins (18, 19, 20, 21). WAVE proteins are held inactive in the WAVE Regulatory Complex (WRC) (22, 23): their N-terminal WAVE Homology Domain (WHD) embeds the WAVE subunit into the WRC, and their C-terminal VCA domain that activates the Arp2/3 complex is masked at the surface of the complex (24). Activation of WAVE and N-WASP is thought to involve a conformational change that exposes their VCA (24, 25, 26). WAVE and N-WASP complexes have complex relationships: depending on the cell system studied, they collaborate for cell migration, compensate for one another or even induce new emerging functions such as the ability of cells to invade 3D extracellular matrices (27, 28, 29, 30). Recently, the WAVE VCA in the WRC was shown to be dispensable for cell migration, provided that there are a WRC and N-WASP to compensate (31).

Like WAVE proteins, NHS family proteins possess an N-terminal WAVE Homology Domain (WHD) and when examined, it was found that NHS family proteins interact with WRC subunits (16, 32, 33). There are several ways for NHS family proteins to interact with WAVE complex subunits. The ABI1 subunit of the WRC can bind to NHS family proteins through its SH3 domain (32, 34). We also demonstrated that NHSL1 assembles an alternative complex that we have named the WAVE Shell Complex (WSC), where NHSL1 replaces the WAVE subunit through its WHD domain (35). In contrast, the WHD of NHSL3 does not enable the assembly of a WSC (34). The NHSL1-containing WSC mediates persistence of single cell migration through a positive feedback loop that sustains branched actin polymerization at the cell front (35).

The capacity of the founding member of the family, NHS, to form a WSC has not been directly examined, and the function of NHS has remained elusive. Since NHS knockdown was shown to increase cell spreading, which is mediated by lamellipodia (16), we examined cell migration in cell lines where the *NHS* gene has been inactivated. We report here that NHS promotes persistent migration of single cells and that this activity requires both the N- and C-terminal regions of the protein. We also found that NHS forms a WSC, like NHSL1 and unlike NHSL3, and that the NHS-containing WSC is necessary for the ABI1 subunit to interact with the N-WASP/WIPF2 complex.

## Results

### Migration phenotype associated with NHS KO

To study the function of the *NHS* gene, we generated KO cells using CRISPR/Cas9 in human untransformed mammary epithelial cell lines that have been previously characterized for the role of NHSL1 and NHSL3 in cell migration (34, 35). We obtained KO clones from both MCF10A and hTERT-HME1 cell lines using two gRNAs that target *NHS* simultaneously. As expected, all *NHS* KO clones carried a biallelic deletion of 52 base pairs corresponding to the two gRNA-induced cuts (Fig. S1). To rescue the single clone obtained in MCF10A, we first studied the NHS isoforms expressed in this cell line and identified two isoforms that differ only by their first exon due to alternative promoters.

These isoforms correspond exactly to the originally described NHS-A and NHS-1A isoforms (13), but this nomenclature was later applied to isoforms that were not exactly the same (3, 16), creating ambiguity. We therefore define isoform 1 (i1, 1651 amino-acids) as the largest one that contains a WAVE homology domain (WHD) in its N-terminus, whereas isoform two (i2, 1474 amino acids) does not (Fig. S2). We obtained fluorescent puromycin-resistant clones using an efficient integrative plasmid that generates Flag-GFP fusion proteins expressed from a so-called safe-harbor locus. The green fluorescence was weak with both NHS fusion proteins but not with Flag-GFP alone.

**Figure 1:**
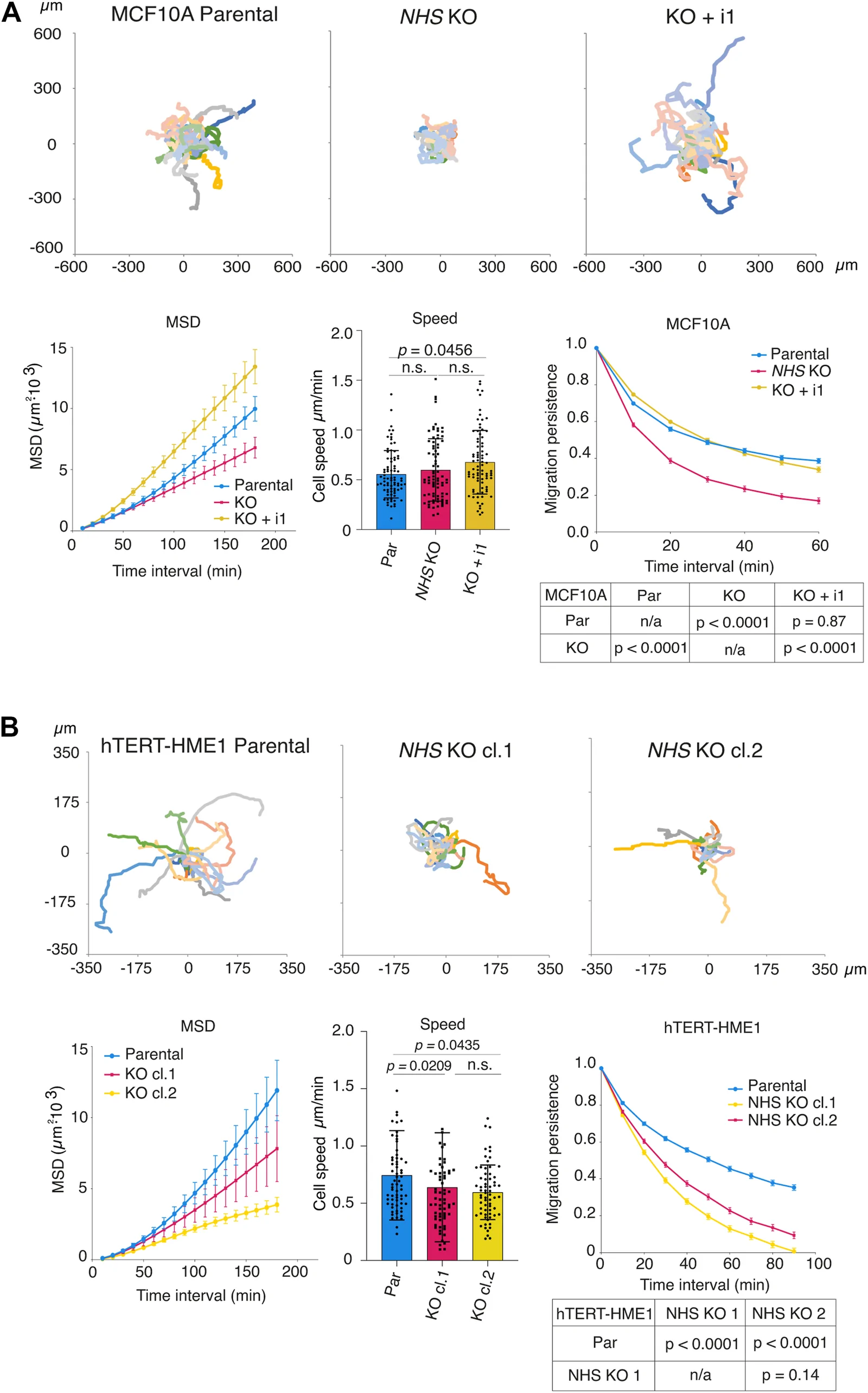
**The Nance-Horan Syndrome (NHS) Protein is required for the persistence of cell migration.** MCF10A and hTERT-HME1 cell lines were knocked out for the *NHS* gene. A derivative expressing full-length isoform 1 of NHS was derived from the single MCF10A *NHS* KO clone. All these cell lines were assayed for random migration of single cells. *A*, trajectories of MCF10A cells and extraction of their migration parameters: speed, migration persistence and Mean Square Displacement (MSD). n = 90 cell. *B*, same experiment with the hTERT-HME1 cell line and two independent *NHS* KO derivatives, n = 66 cells. cl stands for clone. mean ± SEM for MSD and persistence; mean ± SD for speed. In each cell line, three biological replicates gave the same results. Speed averages per cell were compared across conditions by the Kruskal–Wallis test followed by Dunn's *post hoc* test with Holm correction for multiple comparisons. Migration persistences were compared by a *t* test on the contrast coefficient of a nonlinear mixed-effects model with condition as a fixed effect and cell as a random effect; *p*-values were adjusted for multiple comparisons using the Benjamini-Hochberg procedure.

In a 2D migration assay of single cells, *NHS* KO gave a pronounced phenotype that was rescued by stable expression of NHS i1. Indeed, when MCF10A parental cells were compared with *NHS* KO and with KO cells expressing Flag-GFP-NHS i1, cell trajectories, registered to the same starting point, explored a limited territory in KO cells compared with parental cells and NHS i1 rescued this effect (Fig. 1*A*, Movie S1). The area explored by cells is captured by Mean Square Displacement (MSD), which depends on both speed and migration persistence. Speed is not significantly affected in *NHS* KO, but in this random migration assay, migration persistence is. Migration persistence was quantified using the directional autocorrelation function, *i.e.* the decay in migration orientation over time, which is fitted by an exponential nonlinear mixed-effects model for statistical analysis (see Experimental procedures for details). This read-out, used throughout the manuscript, reflects how long a cell sustains the migration direction, once it has polarized. Migration persistence is significantly decreased in *NHS* KO compared with parental cells, and fully rescued by the expression of i1 in the KO genetic context. A small, but significant, increase in speed explains the increased MSD in the rescued cell line compared with parental cells.

To confirm the role of NHS in migration persistence, we turned to the hTERT-HME1 cell line and the *NHS* KO clones we had isolated. In the same random migration assay, *NHS* KO cells were also less persistent than parental cells, whereas speed was not significantly affected (Fig. 1*B* and Movie S2). Together these results indicate that NHS primarily controls migration persistence, the migration parameter that we previously showed to be tightly controlled by WAVE and Arp2/3 complexes (35, 36, 37).

### Partial control over migration persistence exerted by mutated NHS

**Figure 2:**
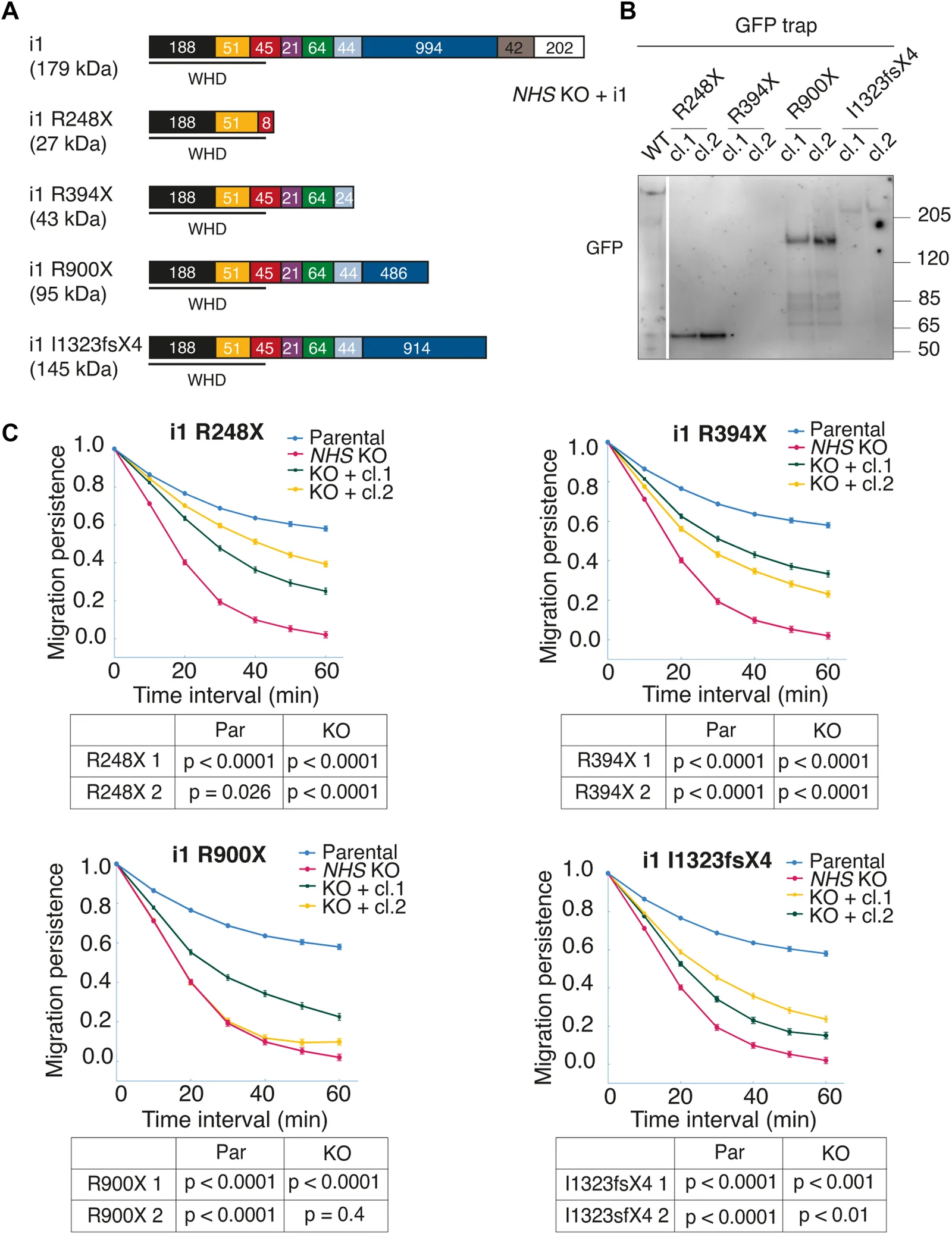
**Truncated forms of NHS found in patients partially rescue cell migration.***A*, mutations found in families affected with the Nance-Horan Syndrome were introduced into the cDNA of NHS isoform 1 (numbering in amino-acids). *B*, expression of wild type and mutant cDNA in N-terminal fusion with Flag-GFP. Stable clones expressing Flag-GFP tagged wild type i1 or mutated derivatives were derived from the *NHS* KO MCF10A clone and analyzed by GFP trap precipitation followed by GFP Western blot. *C*, migration persistence of the different clones. cl. stands for clone. Parental and *NHS* KO curves of all four graphs are the same for reference. n = 79 cells, mean ± SEM. Three biological replicates gave the same results. Migration persistences were compared by a *t* test on the contrast coefficient of a nonlinear mixed-effects model with condition as a fixed effect and cell as a random effect; *p*-values were adjusted for multiple comparisons using the Benjamini-Hochberg procedure.

If the function of NHS in migration persistence is relevant for the pathophysiology of Nance-Horan syndrome, then mutations affecting families displaying this syndrome should also alter migration persistence. We introduced four different mutations found in NHS families into i1 cDNA (Fig. 2*A*): C742T (**R248X**) (7, 9, 14); C1180T (**R394X**), previously referred to as C1117T (R373X) before the short exon 4 of 63 nucleotides was recognized (6, 14); C2698T (**R900X**), previously C2635T (R879X) (6, 14); C3971del11bp (**I1323fsX4**) previously C3908del11bp (14, 38). We then expressed in the *NHS* KO MCF10A clone the Flag-GFP-tagged mutated ORFs from the same locus as wild type i1. With this plasmid, which contains a strong promoter, levels of tagged proteins are usually easily detectable in total cellular lysates (34, 35, 37). But this was not the case with NHS. By concentrating Flag-GFP fusion proteins using GFP trap precipitation, we managed, however, to detect the expression of i1 and some mutant forms of i1, but not all of them (Fig. 2*B*).

The shortest construct R248X, roughly corresponding to the WHD of NHS, and R900X were better expressed than wild type. In contrast, the longest mutant construct I1323fsX4 was at the limit of detection, whereas R394X was below the detection limit. To our surprise, all these mutant constructs partially rescued migration persistence, despite their varied lengths and expression levels (Figs. 2*C* and S3). Even undetectable levels of the variant of R394X rescued as efficiently as more abundant mutant forms of NHS. These results suggest that a small, undetectable amount of NHS is required to control cell migration. Surprisingly, the only clone that did not rescue migration persistence at all was R900X clone two, which expresses more NHS R900X than clone 1, which partially rescued the KO phenotype. The fact that all four tested mutations partly impaired migration persistence suggests that this function of NHS is relevant for NHS pathophysiology. The varying but overly low level of expression of NHS and its derivatives suggests that NHS expression might be controlled in complex ways.

### The NHS-containing WAVE Shell Complex (WSC)

**Figure 3:**
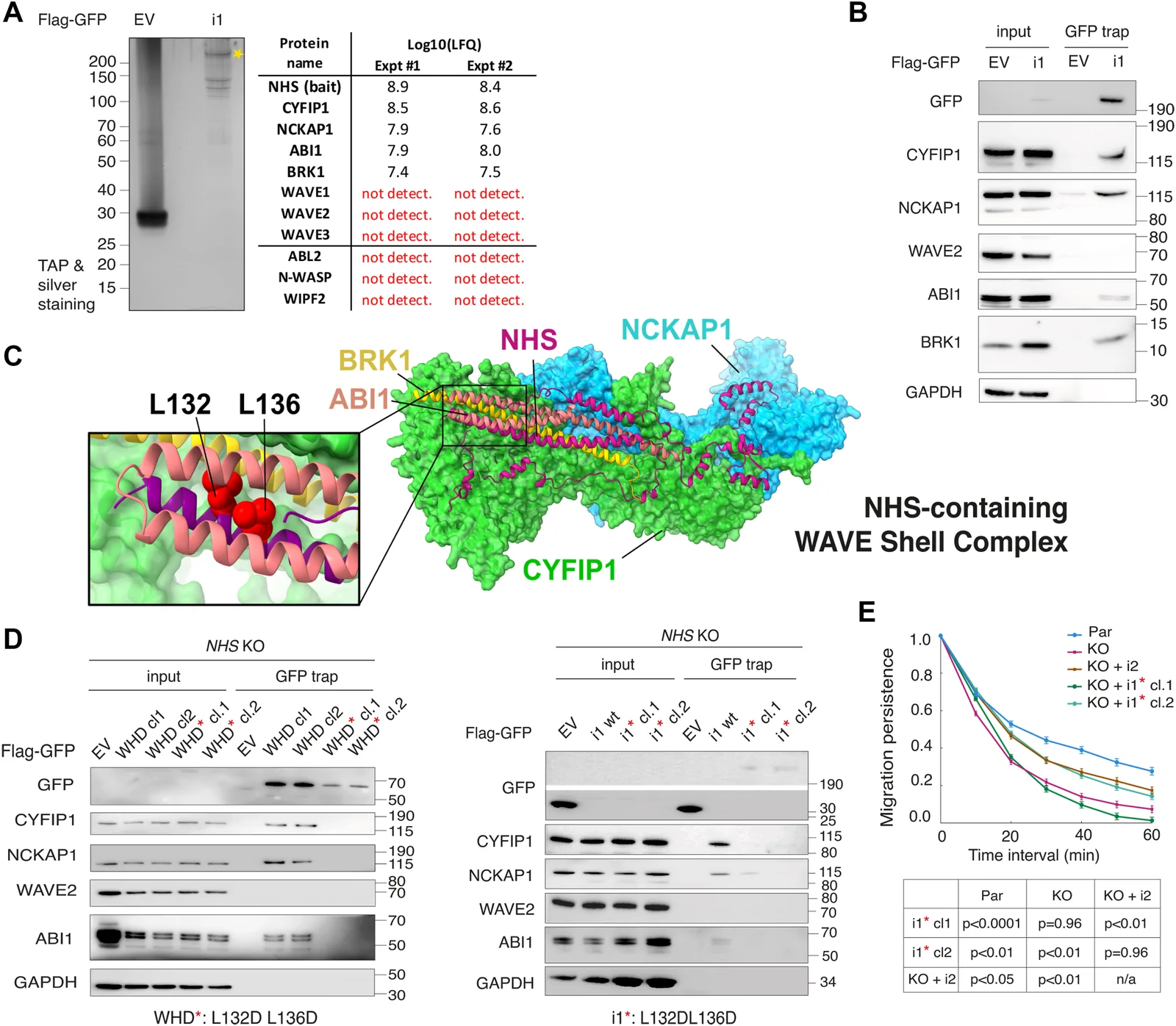
**NHS forms a WAVE Shell Complex.***A*, TAP of NHS isoform 1. Stable MCF10A cells expressing Flag-GFP-NHS isoform 1 or Flag-GFP alone (empty vector, EV) were lysed and subjected to two sequential precipitations targeting Flag and then GFP. The position of the bait is indicated by a *yellow star*. Mass spectrometry was used to identify partners and evaluate their abundance. The Log_10_ of LFQ of the bait, of WAVE Regulatory Complex subunits and of WAVE complex partners is reported in the table. not detect.: not detected. *B*, confirmation of a WSC by Western blot after GFP trap precipitation of Flag-GFP-ABI1. *C*, structural model highlighting NHS interactions within the WSC. AlphaFold3 prediction of the NHS-WSC complex showing NHS (*magenta*) positioned along the BRK1 (*gold*)-ABI1 (*salmon*) coiled-coil core and extending across CYFIP1 (*green*) and NCKAP1 (*cyan*). The inset provides a close-up view of the ABI1-BRK1-NHS predicted coiled-coil, highlighting NHS residues that pack against the interface, including the positions of L132 and L136 (*red spheres*). The magnified view emphasizes the predicted hydrophobic contacts stabilizing NHS association with the WSC core. NHS and ABI1 are shown only for the regions that AlphaFold3 predicted to assemble with the other subunits at high confidence (NHS: 14-34, 123-233, 254-406; ABI1: 1-113). A more detailed analysis of the confidence regions is provided in Fig. S3 and the complete structural model including all the high and low confidence regions can be obtained at https://modelarchive.org/doi/10.5452/ma-1dgvq. *D*, sStable clones expressing Flag-GFP tagged WHD or WHD∗ (L132D L136D), or Flag-GFP tagged full length i1 or i1∗ (L132D L136D) were derived from *NHS* KO cells. Flag-GFP fusion proteins were precipitated with GFP trap beads and precipitates were analyzed by Western blots with the indicated antibodies. *E*, migration persistence of parental MCF10A, *NHS* KO cells and KO clones stably expressing full-length i2 or i1 containing the two L132D and L136D (∗). n = 69 cells, mean ± SEM. Three biological replicates gave the same results. Migration persistences were compared by a *t* test on the contrast coefficient of a nonlinear mixed-effects model with condition as a fixed effect and cell as a random effect; *p*-values were adjusted for multiple comparisons using the Benjamini-Hochberg procedure. cl. stands for clone.

To gain insights into the mechanism by which NHS exerts its control over migration persistence, we performed Tandem Affinity Purification (TAP) of NHS i1 from a stable MCF10A cell line expressing Flag-GFP-NHS i1 or Flag-GFP as a control (Fig. 3*A*). Using mass spectrometry and Label-Free Quantification (LFQ), we were able to identify and quantify many proteins specifically associated with NHS i1 (Table S1). The most abundant ones were the WAVE complex subunits, CYFIP1, NCKAP1, ABI1 and BRK1, but all three WAVE proteins were below the detection limit. We confirmed by GFP trap precipitation followed by Western blot that CYFIP1, NCKAP1, ABI1 and BRK1, but not WAVE2, were associated with NHS (Fig. 3*B*). These results suggest that NHS assembles a WAVE Shell Complex (WSC) like NHSL1 (35).

Using AlphaFold3, we generated a high-confidence structural model of the NHS-containing WSC where NHS interacted with CYFIP1, NCKAP1, BRK1 and ABI1 subunits (Figs. 3*C* and S4). Core to this interaction is the WHD of NHS, especially residues 123 to 233 that form a triple coiled-coil with BRK1 and ABI1, as does the N-terminal helix of the WAVE subunit in the case of the WRC (24, 39). The NHS-containing WSC is thus organized along the same principle as NHSL1-containing WSC (35), but the interaction of NHS with WAVE complex subunits is more extended than was that of NHSL1, since residues 254 to 406 of NHS, following the WHD, also contributed to the interface. We then attempted to design NHS mutations that would prevent its ability to form the WSC. We identified two leucine residues L132 and L136 in the WHD of NHS that appeared critical in the structural model to form the heterotrimeric coiled-coil where NHS, ABI1 and BRK1 contribute one strand each. We mutated these two leucines into charged aspartic acids (L132D L136D) in the WHD and isolated stable clones expressing either wild-type or mutant WHD as Flag-GFP fusion proteins. Upon GFP trap precipitation, we found that the two mutations indeed abolished the interaction with WAVE complex subunits and rendered the WHD less stable than its wild type counterpart (Fig. 3*D*).

The same two mutations were then introduced into full-length NHS i1, and stable *NHS* KO clones expressing this mutated form of i1 were isolated and compared with a clone that expresses i1. In the context of full-length i1, the two mutations also decreased the amount of WAVE complex subunits, confirming that the NHS-containing WSC is the major mode of binding between NHS and WAVE complex subunits (Figs. 3*D* and S5). We then compared i1 containing the WHD-impairing mutations to i2, which naturally does not contain a WHD. We did not manage to detect the expression of tagged i2 even after GFP precipitation. Nevertheless, i2 expression partially rescued the defective migration persistence of *NHS* KO cells. In the migration assay, the two clones expressing i1 containing L132D L136D mutations partially rescued migration persistence of the *NHS* KO, like the one expressing i2, albeit to a different extent (Figs. 3*E* and S6). These results suggest that the ability of NHS i1 to form a WSC through its WHD accounts in part for its ability to control migration persistence.

### The NHS-containing WSC enables an interaction with the N-WASP/WIPF2 complex

**Figure 4:**
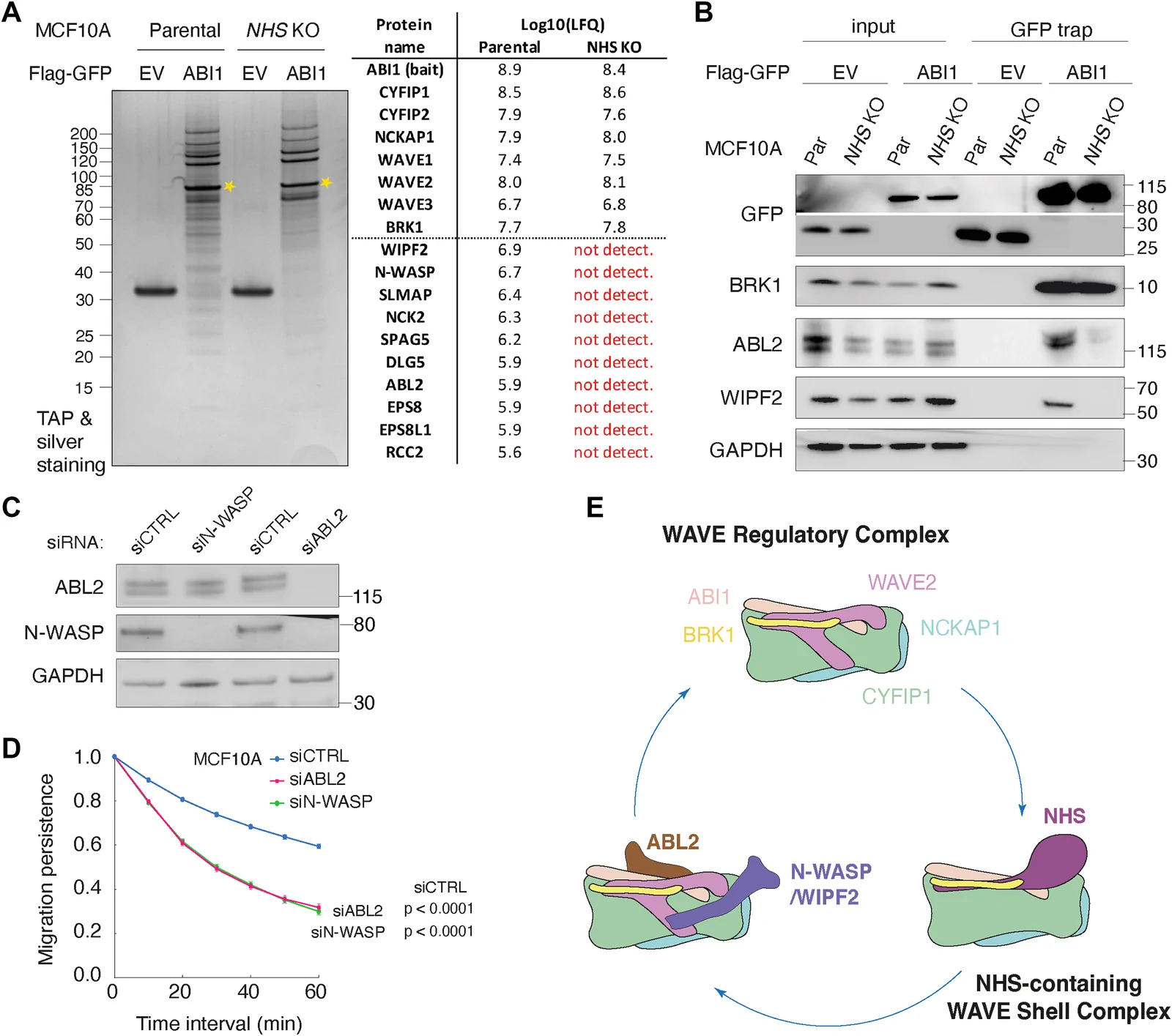
**The WAVE Shell Complex containing NHS is required for ABI1 to interact with the N-WASP/WIPF2 complex.***A*, TAP of ABI1. Parental or *NHS* KO MCF10A cells stably expressing Flag-GFP-ABI1 or Flag-GFP (empty vector, EV) were lysed and subjected to two sequential precipitations targeting first Flag and then GFP. The position of the bait is indicated by a *yellow star*. Mass spectrometry was used to identify partners and evaluate their abundance. not detect.: not detected. The Log_10_ of LFQ of WAVE Regulatory Complex subunits is reported in the table, together with that of partners, which are associated with ABI1 in parental cells, but not in *NHS* KO cells. *B*, confirmation using GFP precipitations and Western blots. *C*, MCF10A cells were transfected with pools of siRNAs targeting N-WASP or ABL2 or non-targeting siRNAs as a control. Three days after transfection, cell lysates were analyzed by Western blot using ABL2, N-WASP or GAPDH antibodies. *D*, migration persistence of N-WASP and ABL2 depleted cells. n = 94 cells, mean ± SEM. Three biological replicates gave the same results. Migration persistences were compared by a *t* test on the contrast coefficient of a nonlinear mixed-effects model with condition as a fixed effect and cell as a random effect; *p*-values were adjusted for multiple comparisons using the Benjamini-Hochberg procedure. *E*, speculative model. WRC would be converted into a WSC, where NHS replaces WAVE2. The NHS-containing WSC would be a prerequisite for ABI1 to bind ABL2 and N-WASP/WIPF2. This model is in line with the facts that NHS is required for ABI1 to interact with ABL2 and N-WASP, even though the TAP of NHS does not retrieve ABL2 nor N-WASP/WIPF2. Therefore we tend to favor this speculative model, where ABL2 and N-WASP/WIPF2 interact with the WRC rather than with the WSC-containing NHS.

To explore the role of the NHS-containing WSC, we performed TAP of ABI1 in parental and *NHS* KO MCF10A cells using stable Flag-GFP-ABI1-expressing clones in the two genetic contexts (Fig. 4*A*). We then identified partners by mass spectrometry and calculated their amounts by LFQ. Out of the hundreds of proteins identified as potential ABI1 partners, only 17 proteins were differentially associated with ABI1 when the *NHS* gene was inactivated or not (Table S2). Among the differential proteins, many proteins were found to specifically associate with ABI1 in parental cells, but not in *NHS* KO cells, and most of these were reported to regulate actin polymerization and cell migration. The most abundant ones were WIPF2, also known as WIRE or WICH (40), and N-WASP, also known as WASL (41), which form a heterodimeric complex (42, 43, 44). The other ones in decreasing order of abundance were SLMAP (45), NCK2 (46), SPAG5 (47, 48), DLG5 (49, 50), ABL2 (51, 52), EPS8 and EPS8L1 (53, 54, 55), and finally RCC2 (56, 57).

We then examined the role in our migration assay of the N-WASP NPF and of ABL2 that promotes branched actin polymerization in collaboration with NPFs (51). Using siRNAs, we depleted MCF10A cells from N-WASP and ABL2 and found that ABL2 depletion resulted in N-WASP depletion, comparable to a direct targeting of N-WASP (Fig. 4*B*). In line with this result, N-WASP- and ABL2-depleted cells were both similarly impaired in migration persistence (Figs. 4, *C* and *D*, S6). Together, these results suggest that the promotion by NHS of the interactions between ABI1 and ABL2, and between ABI1 and the N-WASP/WIPF2 complex, can contribute to the migration-promoting role of NHS.

## Discussion

We report here that NHS regulates cell migration, an important point that was not previously established, even if this result is in line with previous findings that NHS inhibits cell spreading and interacts with WRC subunits (16). In MCF10A and hTERT-HME1 cells, NHS specifically promotes migration persistence, the precise parameter of cell migration that depends on the RAC1-WAVE-Arp2/3 pathway (35, 37). In most cells of the organism and most cell lines, expression levels of NHS are very low (2). That is also the case in the immortalized cell lines of the human mammary gland that we studied here, and where we were unable to detect the NHS protein using Western blots of total cellular lysates. Yet we were able to document a cell migration phenotype upon *NHS* inactivation.

We took advantage of the availability of an assay for NHS function to examine four mutated alleles described in Nance-Horan Syndrome patients. The decreased migration persistence exhibited by *NHS* KO MCF10A cells was fully rescued by the i1 isoform, but only partly by i2 or the truncated i1 produced in patients. This partial rescue is intriguing. It is possible that migration persistence is not the most relevant function of NHS for the Nance-Horan syndrome. It is also possible that morbid alleles of NHS do not correspond to null alleles, but rather to hypomorph alleles, because truly null alleles would be lethal. In all four morbid alleles we tested, the WHD of NHS that we modeled in the NHS-containing WSC is intact even if it does not contain the whole interacting region of NHS. However, several publications reported NHS mutations that directly truncate the WHD that provides the most substantial part of the interaction: Q39X (5), E108X (58), R134fs (2), Q158X (7). These observations raise the question of what would be the minimal N-terminal part of NHS that partially rescues migration persistence if not the WHD.

Our results clearly indicate that NHS assembles a WSC, as we previously reported for NHSL1 but not for NHSL3 (Fig. S7) (34, 35). For NHSL1, the WSC was only one of the two ways of interacting with WAVE complex subunits (32), and we had to use PPP2R1A, the specific partner of the NHSL1-containing WSC, to purify it by affinity (35). For NHS, the WSC seems to be the major mode of interaction with WAVE complex subunits, since WAVE NPFs are below the detection limit of Western blot and mass spectrometry in NHS immunoprecipitates from MCF10A cells. The NHS-containing WSC is also required, like the NHSL1-containing WSC, to recruit specific partners.

By comparing ABI1 TAP from parental MCF10A and *NHS* KO cells, we found that the N-WASP/WIPF2 complex and a handful of other proteins are specifically associated with ABI1 when NHS is present. Most of these partners are involved in actin polymerization and cell migration, showing the remarkable specificity of such quantitative and differential proteomics screen. These partners should now be investigated together as a network of proteins. For example, the dependence on ABL2 for N-WASP stability was to our knowledge not previously reported. Surprisingly, N-WASP/WIPF2 and ABL2, which are absent in ABI1 TAP when NHS is inactivated, do not appear in the NHS TAP, indicating that they do not interact with the NHS-containing WSC, despite their requirement for NHS. Therefore we speculate that N-WASP/WIPF2 and ABL2 interact rather with the WRC and that the NHS-containing WSC is a prerequisite step for their interaction (Fig. 4*E*). But alternative models are possible. ABI1 could interact on its own with its partners, since the ABI1/N-WASP interaction has been detected *in vitro* with purified proteins (59). All the partners that bind ABI1 only when NHS is present may not even bind the same structure of ABI1 nor at the same moment, even if they are similarly involved in actin polymerization and cell migration.

In conclusion, our study reports the existence of an NHS-containing WSC and the recruitment of the N-WASP/WIPF2 complex by ABI1 that depends on NHS, but that is likely not mediated by the NHS-containing WSC. The existence of several alternatives to the WRC in the forms of NHS- and NHSL1-containing WSCs, the activation of the WRC through a WAVE conformational change or the putative exchange of WAVE subunits for NHS family proteins, the potential assembly of NPF super-complexes illustrate the urgent need to re-evaluate what we believe we understand about the generation of branched actin networks and cell migration and to incorporate this complexity in an ordered and coherent molecular activation pathway. The quantitative and differential proteomics screens combining TAP with different genetic contexts, as used here, probably paves the way, or at least a way, to tackle these challenging cell biology questions in the future.

## Experimental procedures

### Cell culture

MCF10A cells were grown in DMEM/F12 medium supplemented with 5% horse serum, 20 ng/ml epidermal growth factor, 10 μg/ml insulin, 100 ng/ml cholera toxin, 500 ng/ml hydrocortisone and 100 U/ml penicillin/streptomycin. The MCF10A cell line was from the collection of breast cell lines organized by Thierry Dubois (Institut Curie, Paris). hTERT-HME1 cells from ATCC (reference ME16C) were grown in RPMI1640 medium, with 10% FBS and 100 U/ml penicillin/streptomycin. Media and supplements were from Life Technologies and Sigma. Cells were incubated at 37 °C in 5% CO2. Cells were passaged every 3 days.

### Plasmids, transfection and isolation of stable cell lines

Flag-GFP-tagged NHS and derivatives were expressed from a home-made vector, MXS AAVS1L SA2A Puro bGHpA EF1Flag GFP Blue SV40pA AAVS1R that was previously described (Molinie *et al.*, 2019). Full-length ORFs of NHS isoforms i1 and i2 were assembled from cDNA amplified from MCF10A cells and inserted into the vector between FseI and AscI sites. To this end, total RNA was extracted using NucleoSpin RNA Plus Kit (Macherey-Nagel). To generate cDNA, 1 μg of total RNA and SuperScript III first strand synthesis for RT-PCR kit (Invitrogen) was used. Site-directed mutagenesis was performed using the QuikChange Lightning kit (Agilent 210519). The WHD (1–278) is naturally extremely GC-rich which renders it difficult to go through by PCR. We worked with a GC-reduced WHD of NHS obtained using synthesis of a gene containing synonymous substitutions. Sequence is available upon request. The previously described plasmid expressing Flag-GFP-ABI1 was used to transfect NHS KO MCF10A cells (35).

Transfections were performed using Lipofectamine 3000 (Invitrogen). To obtain stable cell lines in MCF10A, MXS AAVS1 vectors were co-transfected with two TALEN constructs (Addgene #59025 and 59026) inducing a double-strand break at the AAVS1 locus (60). Cells were selected with 0.5 μg/ml puromycin (Invivogen). Single clones were picked using cloning rings, expanded and analyzed by GFP immunoprecipitations.

### Knock-out and knock-down

MCF10A and hTERT-HME1 knockout cell lines were generated using the CRISPR/Cas9 system with reagents from ThermoFischer Scientific. Cells were transfected with two gRNAs (CRISPR840734_SGM: CTTACTCTCTATGTCCAGGT and CRISPR840727_SGM: GGAGGAAGATGTTGCGCTGC) and the purified Cas9 protein (A36498) using Lipofectamine CRISPRMAX (CMAX00008). After 2 days, cells were diluted to 0.8 cell/well in 96-well plates. Single clones were analyzed using Phire Tissue Direct PCR Master Mix (Thermo Fisher Scientific F170S) and the primers AGACGGTTGGTGTCTTGGAG and CTCCGAGTTGTCTCCCGCAA. The putative KO clones displaying the expected deletion were all confirmed by sequencing.

For knock-down, MCF10A cells were transfected with 20 nM siRNAs using Lipofectamine RNAiMAX (Invitrogen). siRNAs were Dharmacon ON-TARGET SMART Pools targeting N-WASP, ABL2 or non-targeting as a control (L-006444-00-0005; L-003101-00-0005 and D-001810-10-20, respectively). Cells were analyzed 3 days after transfection.

### Antibodies

The antibodies used were anti-GFP (Roche, 11814460001, 1:1000), anti-GAPDH (Thermo Fisher Scientific, AM4300, 1:4000); anti-NCKAP1 (Bethyl Laboratories, A305-178A, 1:3000); Anti-NWASP antibody (Thermo Fisher Scientific 14306-1-AP; 1:1000, validated using siRNAs in Fig. 4*C*), anti-WIPF2 (Ozyme, HPA024467, 1:1000) and anti-ABL2 (Thermo Fisher Scientific 17693-1-AP; 1:1000, validated using siRNAs in Fig. 4*C*); home-made CYFIP1, ABI1, WAVE2 antibodies (61), and BRK1 mAb (39) were previously described.

### Western blots

Cells were lysed in RIPA buffer (HEPES 50 mM, EDTA 10 mM, SDS 0.1%, NP-40 1%, DOC 0.5%, NaCl 15 mM, pH7.4) supplemented with protease inhibitors (Roche), the lysates were clarified by centrifugation at 14,000 rpm for 15 min and subjected to SDS–PAGE using NuPAGE 4 to 12% Bis-Tris or 3 to 8% Tris-Acetate gels (Life Technologies). Pieces of nitrocellulose membranes were cut and incubated with different primary antibodies, HRP-conjugated secondary antibodies (Sigma) and developed with SuperSignal West Femto Substrate (Thermo Fisher Scientific) and ImageQuant 800 (Cytiva).

### Immunoprecipitations and TAP

Cells stably expressing Flag-GFP-ABI1 (35) or Flag-GFP-NHS i1 were lysed with XB-NP40 buffer (50 mM HEPES, 50 mM KCl, 1% NP-40, 10 mM EDTA, pH 7.7) supplemented with protease inhibitors at 4 °C for 30 min. The lysates were clarified by centrifugation at 13,000 rpm for 15 min. Clarified cell extracts were incubated with Flag-M2 beads (Sigma) at 4 °C for 4 h. Flag-M2 beads were washed with XB-NP40 buffer and eluted with 0.5 mg/ml Flag peptide (Sigma) in XB (50 mM HEPES, 50 mM KCl, 10 mM EDTA, pH 7.7) overnight at 4 °C. Flag elutions were collected and incubated with GFP-trap beads (Chromotek) at 4 °C for 1 h. The GFP-trap beads were washed with XB-NP40 buffer. 20% of the beads were subjected to SDS–PAGE for Western blot or silver staining (SilverQuest Silver Staining Kit, Thermo Fisher Scientific). In all, 80% of the beads were analyzed by mass spectrometry.

### Mass spectrometry

The samples are first reduced with 10 mM DTT at 56 °C for 30 min, then alkylated with 50 mM iodoacetamide at room temperature for 20 min. They are subsequently digested overnight at 37 °C using a trypsin/Lys-C mix (0.5 μg). The resulting peptides are desalted and concentrated, then reconstituted in 15 μl of injection buffer A’’(0.1% TFA/Acetonitrile 98:2 (v/v)). For each fraction, 2 μl of sample was concentrated on a C18 cartridge (Dionex Acclaim PepMap100, 5 μm, 300 μm i.d. × 5 mm) and eluted on a capillary reverse-phase column (nanoEaze M/Z Peptide CSH C18, 1.8 μm, 75 μm i.d. × 25 cm) at 300 nl/min, with a gradient of 2% to 38% of buffer B in 60 min (buffer A: 0.1% aq. Formic Acid/Acetonitrile 98:2 (v/v); buffer B: 0.1% aq. Formic Acid/Acetonitrile 10:90 (v/v)).

For the TAP of NHS, the eluate was injected into an Orbitrap Eclipse Tribrid mass spectrometer (Orbitrap Eclipse Tribrid, ThermoFisher Scientific). MS1 spectra were acquired in the Orbitrap: 1 survey MS scan (375–1500 m/z; resolution 120,000) with dynamic exclusion of 40 s and MS2 spectra were collected in the linear ion trap (centroid data). For the TAP of ABI1, the eluate was injected into a quadrupole-Orbitrap mass spectrometer (Q Exactive HF, ThermoFisher Scientific) using a Top 20 data-dependent acquisition MS experiment: 1 survey MS scan (400–2000 m/z; resolution 70,000) followed by 20 MS/MS scans on the 20 most intense precursors (dynamic exclusion of 30 s, resolution 17,500).

Protein identification was performed with MaxQuant search engine v.2.0.3.0 (62) against the human Swiss-Prot database (updated in 01/2025) with the following parameters: methionine oxidation, acetylation (N-ter) and cysteine carbamidomethylation as variable modifications, first search error tolerance 20 ppm, main error tolerance 6 ppm, MS/MS error tolerance 20 ppm, FDR 1%. Quantification was performed with the LFQ normalization mode using at least two razor or unique peptide per protein. Quantities were estimated using LFQ intensities. Proteins detected in at least two of 3 biological replicates, and not detected in any of the control samples, were considered specific partners.

### Cell migration

Cell migration assays were performed in μ-Slide eight-well dishes (#80826, Ibidi), coated with 20 μg/ml Fibronectin (Sigma). Videomicroscopy was performed 24 h after seeding using an inverted Axio Observer microscope (Zeiss) equipped with a Pecon Zeiss incubator XL multi S1 RED LS (Heating Unit XL S, Temperature module, CO2 module, Heating Insert PS and CO2 cover), a definite focus module and a Hamamatsu camera C10600 Orca-R2. Images were acquired every 10 min for 24 h with a × 10 objective. Individual cells were tracked using the Manual Tracking plug-in in the ImageJ software. The DiPer software was used to represent cell trajectories (Plot_at_origin.txt) and analyze the single cell migration parameters: MSD (MSD.txt) and migration persistence (Autocorrel.txt) (63). Migration persistence represents the direction autocorrelation, characterizing the orientation of two displacement vectors, *i.e.* the cosine of the angle they form, as a function of the time interval that separates these displacement vectors. The outputs of DiPer are mean ± SEM.

### Structural modeling

Structural modeling of the human multimeric complex formed by NHS (UniProt Q6T4R5), ABI1 (Q8IZP0), BRK1 (Q8WUW1), CYFIP1 (Q7L576), and NCKAP1 (Q9Y2A7) was performed using AlphaFold 3 (64). Full-length sequences were provided as input without structural templates, and the prediction was refined through ten recycling iterations. Model confidence was evaluated using AlphaFold 3 scoring metrics, yielding a global ipTM of 0.78, a global pTM of 0.68, and a ranking confidence score of 0.80 with no detectable steric clashes in the modeled structure. Interface confidence was assessed using pairwise ipTM values with highly supported interactions including NHS-BRK1 (0.90), BRK1-CYFIP1 (0.88), CYFIP1-NCKAP1 (0.89), CYFIP1-ABI1 (0.85) and BRK1-ABI1 (0.84). Predicted alignment error (PAE) values between chains were low in the regions shown in Figure 3*C* and reported in Fig. S4. At the interfaces of the complex, high-confidence predictions were restricted for NHS to residues 1 to 233 and 254 to 406 and for ABI1 to residues 1 to 133.

### Statistical analysis

Cell speed was averaged for each cell, and the averages for each cell were compared across conditions by the Kruskal-Wallis test followed by Dunn's *post hoc* test with Holm correction for multiple comparisons. This analysis was performed using GraphPad Prism. Statistics comparing cell speeds in each condition are available in Table S3.

Directional persistence was quantified as the direction autocorrelation of each cell's trajectory (63). For each condition, the decay of the autocorrelation was fitted to A(t) = exp(-t/τ) using a nonlinear mixed-effects model implemented with the nlme package (65) in R (R Foundation for Statistical Computing, Vienna, Austria), with the decay rate parameterized on a log scale. Genetic condition was entered as a fixed effects on log(1/τ), and a random effect on log(1/τ) was included to account for repeated measurements within cells. Conditions were compared pairwise by fitting, for each pair, a model in which the decay rate (1/τ) was allowed to differ between the two conditions; the *p*-value tests the null hypothesis of equal decay rate (equal persistence time τ). Visual inspection of residual plots and of the distribution of random effects did not reveal any obvious deviations from homoscedasticity or normality. The resulting *p*-values were adjusted for multiple comparisons by the Benjamini-Hochberg (FDR). The analysis was made in Microsoft Excel, Python and R. Statistics comparing migration persistence in each condition are available in Table S4.

Differences were considered significant at confidence levels greater than 95% (*p* < 0.05). Four levels of statistical significance were distinguished: *p* < 0.05; *p* < 0.01; *p* < 0.001; *p* < 0.0001.

No statistical method was used to predetermine sample size. No outlier points were excluded from analyses. The experiments were not randomized. The investigators were not blinded to allocation during experiments and outcome assessment.

## Data availability

The original migration data that support the findings of this study are available in the Source data file.

Raw files of the LC-MSMS analyses and the database researches have been deposited in PRIDE (https://www.ebi.ac.uk/pride/) with the accession number PXD070850 (TAP NHS) and PXD070942 (TAP ABI1).

Coordinates of the NHS-containing WSC were deposited in the ModelArchive server and are available at https://modelarchive.org/doi/10.5452/ma-1dgvq.

## Supporting information

This article contains supporting information (31, 34, 35).

## Conflict of interest

The authors declare that they have no conflicts of interest with the contents of this article.
