# Supplementary material for: The Nance-Horan syndrome protein NHS regulates cell migration persistence by organizing WAVE and N-WASP nucleation-promoting factor complexes
Source: J Biol Chem. 2026 Aug 13;302(10):113443. doi: 10.1016/j.jbc.2026.113443 (PMC13578402; doi:10.1016/j.jbc.2026.113443)
Supplement: Supplementary file 1 [file mmc1.docx]

**SUPPORTING INFORMATION**

**Supplementary Figures ………………………………………… 2**

**Legends to Movies	……………………………………………… 9**

**Figure S1. Characterization of *NHS* KO clones.** **A** Organization of the *NHS* gene. Length of the coding part of the ten exons (from E1 and E1’ to E9) is indicated in nucleotides. **B** PCR on genomic DNA of MCF10A cells and hTERT-HME1 cells and KO derivatives. For MCF10A cells, an heterozygous mutant clone (+/-) and the homozygous mutant clone (-/-) obtained are shown. **C** Sequencing chromatograms of the PCR product derived from genomic DNA prepared from parental MCF10A cells (+/+) and the single KO clone isolated (-/-). All *NHS* KO alleles, the two alleles of the single MCF10A KO clone and the two alleles of each of the two hTERT-HME1 KO clones, correspond to the shown 52 bp deletion in exon 2 imposed by the two gRNA-induced double strand breaks.

**Figure S2. A** Schemes of the two mRNA isoforms expressed in MCF10A cells. They are due to an alternative first exon. The length of coding sequences of exons and isoforms is indicated in nucleotides (nt). Diagnostic RT-PCR products are indicated. **B** Primers used to amplify RT-PCR products. Their expected sizes are indicated in base pairs (bp). **C** RT-PCR of the two mRNA isoforms. **D** Representations of the two protein isoforms. Isoform 2 does not possess a WAVE Homology Domain (WHD). The two isoforms possess predicted ABI1 SH3 binding sites (Asbs) in exon 7 (#1: 845-855 aa; #2: 1051-1062 aa, numbering according to isoform 1).

**Figure S3. Additional migration parameters, related to the experiment displayed in Figure 2C.** Cell trajectories, Mean Square Displacement (MSD) and speed of single cells. The axes of cell trajectories are in µm. Data are presented as mean ± SD for speed and as mean ± SEM for MSD. Cell speed averages were compared across conditions by the Kruskal–Wallis test followed by Dunn's post-hoc test with Holm correction for multiple comparisons. ** P<0.01, *** P<0.001, ns: non significant.

**Figure S4. Confidence assessment of the NHS-containing WSC model, related to the Figure 3C.** **A** AlphaFold3 model of the assembled complex showing NHS (14-34, light pink; 123-233, pink; 254-406, magenta), ABI1 (1-113, salmon), BRK1 (gold), CYFIP1 (green), and NCKAP1 (cyan). **B** PAE (Predicted Alignment Error) heat map for the full complex, with axes colored to indicate residue ranges for each subunit (dashed lines correspond to undisplayed regions due to very low confidence). Blocks of low PAE (from blue to orange) denote highly confident intramolecular and intermolecular contacts, while higher PAE (from yellow to white) indicates regions of lower confidence or flexibility. **C** PAE map for the full complex, as in B, but with gradient of grays to denote prediction confidence and colored blocks along the diagonal indicating the regions shown in the structural model. The full-length structural model and PAE map can be accessed at https://modelarchive.org/doi/10.5452/ma-1dgvq.

**Figure S5. Additional migration parameters, related to the experiment displayed in Figure 3E.** Cell trajectories, Mean Square Displacement (MSD) and speed of single cells. The axes of cell trajectories are in µm. Data are presented as mean ± SD for speed and as mean ± SEM for MSD. Cell speed averages were compared across conditions by the Kruskal–Wallis test followed by Dunn's post-hoc test with Holm correction for multiple comparisons. *** P<0.001, ns: non significant.

**Figure S6. Additional migration parameters, related to the experiment displayed in Figure 4D.** Cell trajectories, Mean Square Displacement (MSD) and speed of single cells. The axes of cell trajectories are in µm. Data are presented as mean ± SD for speed and as mean ± SEM for MSD. Cell speed averages were compared across conditions by the Kruskal–Wallis test followed by Dunn's post-hoc test with Holm correction for multiple comparisons. ns: non significant.

**Figure S7. NHS family proteins with experimentally confirmed binding sites for WAVE complex subunits.** WSC: WAVE Shell Complex. Asbs: ABI1 SH3 binding site. Residue numbering is from the reference isoform of Uniprot: NHS (Q6T4R5-1), NHSL1 (Q5SYE7-1), NHSL2 (Q5HYW2-1) and NHSL3 (Q9P206-1). The NHS-containing WSC is reported in this study. The NHSL1-containing WSC has been reported in (1). The two Asbs of NHSL1, Asbs #1 (KKPPLPPSR) and Asbs #2 (KPKPKVPER) have been reported in (2). The fact that NHSL3 does not form a WSC, but contains an Asbs (SLRKLKRPPPPP) has been reported in (3).

1. Wang, Y., Chiappetta, G., Guérois, R., Liu, Y., Romero, S., Boesch, D. J., Krause, M., Dessalles, C. A., Babataheri, A., Barakat, A. I., Chen, B., Vinh, J., Polesskaya, A., and Gautreau, A. M. (2023) PPP2R1A regulates migration persistence through the NHSL1-containing WAVE Shell Complex. *Nat. Commun.* **14**, 3541

2. Law, A.-L., Jalal, S., Pallett, T., Mosis, F., Guni, A., Brayford, S., Yolland, L., Marcotti, S., Levitt, J. A., Poland, S. P., Rowe-Sampson, M., Jandke, A., Köchl, R., Pula, G., Ameer-Beg, S. M., Stramer, B. M., and Krause, M. (2021) Nance-Horan Syndrome-like 1 protein negatively regulates Scar/WAVE-Arp2/3 activity and inhibits lamellipodia stability and cell migration. *Nat Commun*. **12**, 5687

3. Novikov, N. M., Gao, J., Fokin, A. I., Rocques, N., Chiappetta, G., Rysenkova, K. D., Zea, D. J., Polesskaya, A., Vinh, J., Guerois, R., and Gautreau, A. M. (2025) NHSL3 controls single and collective cell migration through two distinct mechanisms. *Nat. Commun.* **16**, 205

**Legends to Movies**

**Movie S1.**

Parental MCF10A cells, *NHS* KO and *NHS* KO cells stably expressing Flag-GFP-NHS i1 cells are recorded and tracked. Elapsed time is in h and min. Scale bar: 100 μm.

**Movie S2.**

Parental hTERT-HME1 cells and *NHS* KO cells are recorded and tracked. Elapsed time is in h and min. Scale bar: 100 μm.
